# A preliminary exploration on the mechanism of the carbapenem-resistance transformation of *Serratia marcescens in vivo*

**DOI:** 10.1186/s12864-023-09904-2

**Published:** 2024-01-02

**Authors:** Qian Xu, Beiwen Zheng, Kaixuan Li, Ping Shen, Yonghong Xiao

**Affiliations:** 1 Laboratory Medicine Center, Department of Transfusion Medicine, Zhejiang Provincial People’s Hospital (Affiliated People’s Hospital), Hangzhou Medical College, Hangzhou, Zhejiang China; 2 State Key Laboratory for Diagnosis and Treatment of Infectious Diseases, Collaborative Innovation Center for Diagnosis and Treatment of Infectious Diseases, The First Affiliated Hospital, School of Medicine, National Clinical Research Center for Infectious Diseases, Zhejiang University, NO.79 Qingchun Road, Hangzhou, 310003 Zhejiang Province China

**Keywords:** *Serratia marcescens*, Carbapenem-resistance, *bla*_KPC_

## Abstract

**Background:**

The infection of carbapenem-resistant organisms was a huge threat to human health due to their global spread. Dealing with a carbapenem-resistant *Serratia marcescens* (CRSM) infection poses a significant challenge in clinical settings. This study aims to provide insights into strategies for controlling CRSM infection by exploring the transformation mechanism of carbapenem-resistance.

**Methods:**

We used whole genome sequencing (WGS) to investigate the mechanism of carbapenem resistance in 14 *S. marcescens* isolates in vivo. The expression level of related genes and the minimum inhibitory concentration of meropenem (MIC_MEM_) were also evaluated to confirm the mechanism of carbapenem resistance.

**Results:**

Seven groups of *S. marcescens*, each consisting of two strains, were collected from a hospital and displayed a shift in MIC_MEM_ from low to high levels. Homology analysis revealed that the isolates in five groups were significantly different from the remaining two. WGS and experimental evidence indicated that four groups of strains developed carbapenem resistance by acquiring the *bla*_KPC_ (obtaining group), while two groups (persisting group) increased the expression level of the *bla*_KPC_. In contrast, isolates in the last group (missing group) did not carry the *bla*_KPC_. All strains possessed multiple β-lactamase genes, including *bla*_CTX−M−14_, *bla*_SRT−1_, and *bla*_SRT−2_. However, only in the missing group, the carbapenem-resistant strain lost an outer membrane protein-encoding gene, leading to increased *bla*_CTX−M−14_ expression compared to the carbapenem-susceptible strain.

**Conclusion:**

The study findings suggest that *S. marcescens* strains developed diverse carbapenem resistance in vivo through the evolution of drug resistance, rather than through clone replacement. We hypothesize that carbapenem resistance in *S. marcescens* was due to certain clonal types with a distinct mechanism.

**Supplementary Information:**

The online version contains supplementary material available at 10.1186/s12864-023-09904-2.

## Introduction

*Serratia marcescens*, a member of the family Enterobacteriaceae, is an opportunistic pathogen that has recently become commonly associated with hospital-acquired infections. For almost one and a half centuries, it was believed to be a fungus without pathogenicity after it was first observed in 1819 by the Italian Chemistry professor Bartolomeo Bizio [1]. However, in the last two decades, reports of infections from various sites [2–9] have increased. In fact, current emergence of resistant isolates worldwide has narrowed the therapeutic options against this pathogen. A recent systematic review [10] found that *S. marcescens* is resistant to a wide range of antibiotics, including penicillin, cephalosporin, tetracycline, macrolide, nitrofurantoin, and colistin. It also pointed out that carbapenem should be included in the treatment of *S. marcescens* infections. The emergence of carbapenem-resistant strains was a consequence of the excessive use of broad-spectrum antibiotics. Due to the intrinsically resistance of *S. marcescens* to colistin [11], tigecycline is commonly used in anti-infection treatment in China. Due to the different distribution of tigecycline in vivo, the efficacy of specific infection sites is limited. Therefore, the infection of CRSM has become a major challenge in clinical settings. With both the increasing number of infections and the dissemination of carbapenem resistant strains, *S. marcescens* infection has become a global threat to human health.

In recent years, local outbreaks and epidemics of CRSM had occurred in many countries, including Italy, Brazil, South Africa and Argentina [12–15]. In China, CRSM strains were also widely distributed in distinct regions as relevant reports from Zhejiang, Anhui, Jiangsu, Sichuan, Jilin, Guangdong, Ningxia, and other provinces. In Zhejiang province, cases of nosocomial infection caused by CRSM have been increasingly reported, and the main cause of carbapenem-resistance has been identified as the production of carbapenemases, specifically *Klebsiella pneumoniae* carbapenemase (KPC) [16, 17]. Strains that harbor a *bla*_KPC−2_ plasmid show low-level resistance to meropenem (MIC 2–8 µg/ml), as well as resistance to penicillin, cephalosporins, and aztreonam, but remain sensitive to quinolones and aminoglycosides [17]. In our previous study, all CRSM strains collected from a tertiary hospital were resistant to meropenem (MICs between 64 and 512 µg/ml), quinolones, and aminoglycosides because of harboring *bla*_KPC−2_ while one strain was even resistant to tigecycline [18]. It was indicated that CRSM had developed and would continue to undergo changes in drug resistance. Therefore, suitable and prompt approach should be devised and implemented for clinical infection prevention and control.

The development of beta-lactam resistance in *S. marcescens* involves several mechanisms, including the production of beta-lactamases, decreased permeability of the outer membrane, modification of the target penicillin-binding proteins (PBPs), overexpression of active efflux systems, synthesis of aminoglycoside modifying enzymes, and structural alteration of the GyrA protein [19]. Our previous study revealed that carbapenem resistance in CRSM was caused by the production of carbapenemase, a decrease in the permeability of the outer membrane, and the operation of functional efflux pump systems, with KPC production being the most common [18]. The transformation of carbapenem resistance in CRSM is considered as an evolution of drug resistance under the pressure of antibiotics. Since it is not common in clinical practice, there is not enough clinical data to determine the potential risk factors and reveal the exact truth of the transformation. This research aims to investigate the potential carbapenem resistance mechanism of CRSM in vivo by collecting and analyzing duplicated CRSM strains with altered carbapenem resistance from the same patient using whole genome sequencing (WGS).

## Materials and methods

### The strains information

In a tertiary hospital of Hangzhou with a period from 2014 to 2019, 66 non-duplicated CRSM strains were collected from patients during their hospitalization. After confirming the resistance to meropenem by broth dilution test, the transformation of carbapenem resistance was found in seven patients after a search of clinical data. Finally, 14 strains were collected from the seven patients with each two from a same patient. All strains were identified by MALDI-TOF mass spectrometry. During the interval of isolation time, all patients were exposed to invasive operations and received broad-spectrum antibiotics including piperacillin tazobactam, cefoperazone sulbactam and meropenem in prescribed doses. Besides, all strains were hospital acquired except strain 83 and all strains were isolated from sputum except strain 31.

### Antimicrobial susceptibility testing

The minimal inhibitory concentrations (MICs) of meropenem against CRSM strains were determined by the broth microdilution method. Carbapenem resistance was defined as resistance to any carbapenems in accordance with 2020 Clinical and Laboratory Standards Institute guidelines [11]. *Escherichia coli* ATCC 25,922 was used as a quality control strain. The efflux pump inhibitor test was performed with the efflux pump inhibitors including 1-(1-naphthylmethyl)-piperazine (NMP), phenylalanine arginine β-naphthylamide (PAβN) and carbonyl cyanide m-chlorophenylhydrazone (CCCP) [20].

### WGS and homology analysis

All strains were cultured overnight in Mueller-Hinton broth at 37 °C for genomic DNA extraction using the QIAamp DNA Mini Kit (QIAGEN, Germany). All the genomic DNAs were sequenced by using paired-end 500-bp insert libraries on an Illumina HiSeq X Ten instrument. The resulting 150-bp Illumina reads were assembled by CLC Genomics Workbench software with default settings. To obtain complete genome assemblies, four strains (24,26,82,152) from the persisting group were sequenced sicne they were comparable on the Nanopore MinIon platform of Novogene company in China. Antibiotic resistance genes were identified by resFinder3.2 (https://cge.cbs.dtu.dk/services/ResFinder/) with the default threshold. For homology analysis, a local core genome multi-locus sequence typing (cgMLST) scheme was established and a minimum spanning tree was generated as previous indicated [18].

### Quantitative polymerase chain reaction(qPCR) of drug resistant genes

The mRNA expression levels of drug resistant genes including *bla*_KPC−2_, *bla*_CTX−M−14_, *bla*_SRT−1_ and *bla*_SRT−2_ were examined by qPCR. Overnight bacterial cultures were diluted 1/100 in LB broth and grown to log phase at 37 °C with vigorous shaking. Total RNA was harvested by the RNeasy Mini Kit (QIAGEN, Germany). The yield and quality of RNA were determined by Nanodrop 2000 (Thermo, USA). Total RNA was reverse transcribed into cDNA by the Prime Script RT Reagent kit (Takara, China). Quantitative PCR was run on a CFX96 Real-Time PCR Detection System (Thermo, USA) with 40 cycles of 5 s at 95 °C, 30 s at 60 °C after 1 cycle of 30 s at 95 °C. SYBR Premix Ex Taq (Takara, China) was used to quantify the expression of the target gene. The reactions were performed in a volume of 25 µL and the primers used in these experiments are listed in Table 1. The expression of each gene was normalized to that of a housekeeping gene (rpoB). Data were analyzed using the 2^−ΔΔCT^ method. The unpaired t-test was used to compare the expression of drug resistant genes. All *P* values were two-tailed, and p < 0.01 was considered statistically significant.

## Results

### The distribution of clonal types in seven groups

**Fig. 1:**
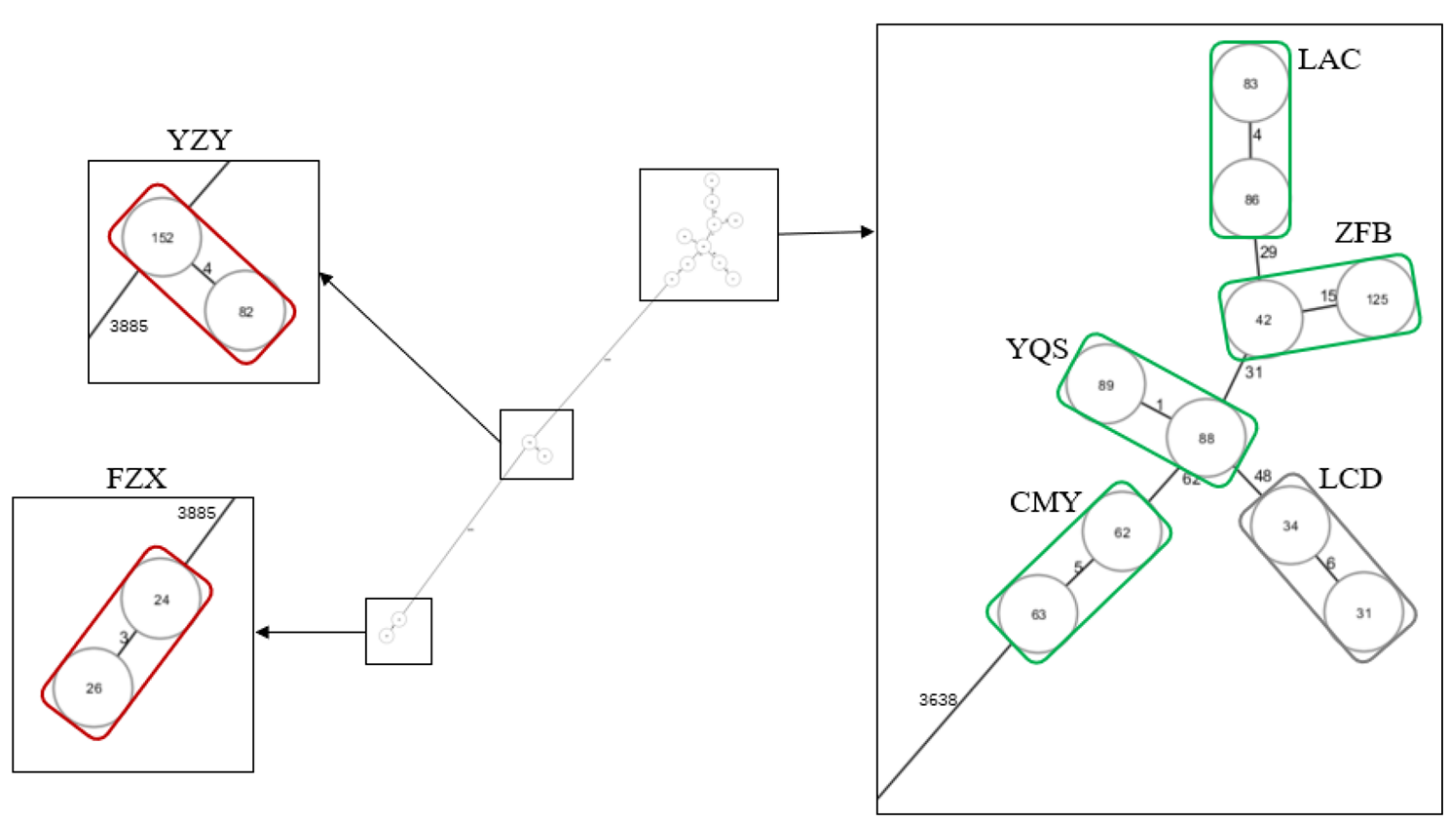
Minimum spanning tree of fourteen strains from seven groups

A total of fourteen strains were collected and classified into seven groups based on the same patient. Based on the presence of *bla*_KPC_, seven groups of strains were further divided into obtaining (LAC, ZFB, YQS, CMY), persisting (YZY, FZX) and missing groups (LCD). Within each group, strains from the same patient exhibited an increasing in MIC_MEM_ by 8 to 1024 times (Table 1). Based on WGS data, a minimum spanning tree was generated, which demonstrated that all strains could be divided into three clonal types. As shown in Fig. 1, five groups (LAC, ZFB, YQS, CMY and LCD) on the right belonged to the same clone type, while group FZX and group YZY on the left belonged to other two different clone types. However, strains within each group were closely related to each other, with the distinct allelic genes differing by less than sixteen.

Seven groups were divided into three clonal types as shown above. The right one including four obtaining groups (in green box) and one missing group (in grey box). The left two were the persisting groups YZY and FZX (in red box), respectively. The group names were next to each box. Numbers next to the lines indicated the distinct allelic genes between two strains.

**Table 1:** The basic characteristics and comparison of drug resistance in seven group strains

Group	Age/sex	Strain NO.	Source	Ward	MIC_MEM_ (µg/ml)	Date	*bla*_KPC_	Other resistant genes*
CMY	62/F	62	sputum	Neurosurgery	0.0625	5/25/2017	-	*bla*_CTX−M−14_, *bla*_SRT−1_, *qnrS1*, *aac(6’)-Ic*
		63	sputum		4	5/31/2017	+	
LAC	66/M	83	sputum	Neurosurgery	0.0625	8/22/2019	-	*bla*_SRT−1,_*qnrS1*, *aac(3)-IId*, *aac(6’)-Ic*, *bla*_LAP−2_
		86	sputum		2	8/30/2019	+	
YQS	50/M	88	sputum	ICU	0.0625	9/11/2019	-	*bla*_CTX−M−14_, *bla*_LAP−2_, *bla*_SRT−1_, *qnrS1*, *aac(6’)-Ic*, *aac(3)-Iid*
		89	sputum		64	9/12/2019	+	
ZFB	60/F	42	sputum	Neurosurgery	0.5	6/21/2017	-	*bla*_CTX−M−14_, *qnrS1*, *bla*_LAP−2_, *bla*_SRT−1_, *aac(6’)-Ic*
		125	sputum		32	7/31/2017	+	
FZX	62/M	24	sputum	ICU	1	7/8/2014	+	*bla*_SRT−1_, *bla*_SRT−2_, *aac(6’)-Ic*
		26	sputum		32	7/15/2014	+	
YZY	89/M	82	sputum	Gerontology	8	8/14/2019	+	*bla*_SRT−1_, *bla*_SRT−2_, *qnrS1, aac(6’)-Ic*
		152	sputum		64	10/17/2019	+	
LCD	58/M	31	blood	Infection	0.5	1/31/2015	-	*bla*_CTX−M−14_, *bla*_LAP−2_, *bla*_SRT−1_, *bla*_OXA−1_, *qnrS1, ARR-3*, *mph(A)*, *aac(6’)-Ib-cr*, *aac(6’)-Ic, aac(3)-Iid, catB3,qacE, aac(6’)-Ib-cr, sul1*
		34	sputum		8	3/1/2015	-	

*: including β-lactam, quinolone and aminoglycoside resistance genes

### The transformation mechanism of carbapenem resistance in obtaining groups

Table 1 reveals that the MIC_MEM_ of strains in obtaining groups (group CMY, LAC, YQS and ZFB) increased by acquiring a *bla*_KPC_ gene. The mRNA expression level of *bla*_KPC_ was detected from none to a normal cycle threshold (CT) value in each group by quantitative polymerase chain reaction (qPCR). The normal CT values detected in group CMY, LAC, YQS and ZFB were 22.68, 17.54, 15.36 and 17.07, respectively. It was observed that the *bla*_KPC_ is located in the IncF type plasmid with two transposase around it, one of which were directly connected with the *bla*_KPC_. However, there was no *bla*_KPC_ gene exist in CRKP, CRAB and CRPA strains which isolated earlier than strain 89 and strain 125 from the same patient (Supplemental Fig. 1).

### The mechanism of carbapenem resistance transformation in persisting groups

**Fig. 2:**
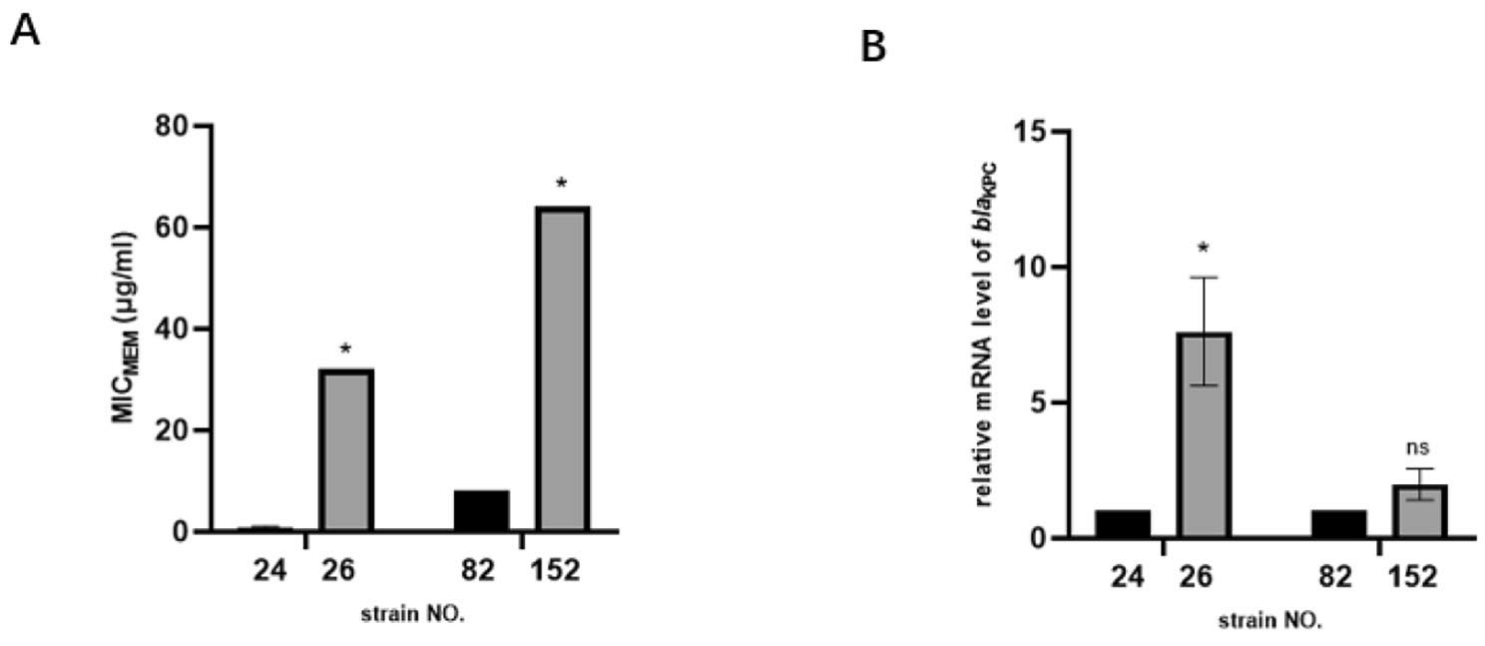
Comparison of relative KPC expression levels in persisting groups. (**A**). The MIC_MEM_ of persisting groups.; (**B**). The relative mRNA expression of *bla*_KPC_ gene in persisting groups MIC_MEM_: minimum inhibitory concentration of meropenem. *:P < 0.01, ns: not significant

In group FZX and group YZY (persisting groups), strains harboring the same *bla*_KPC_ gene with different levels of minimum inhibitory concentration for meropenem (MIC_MEM_) (Fig. 2A). The qPCR results confirmed the findings from the transcriptome sequencing data (data not shown), revealing a nine-fold increase in mRNA expression levels in group FZX and a two-fold increase in group YZY (Fig. 2B). The *bla*_KPC_ genes were all located on the plasmids of the size above ~ 100 kb and belonged to the group incompatibility F. In group FZX, the genetic context of *bla*_KPC_ exhibited 15 different base pairs distributed mostly in *bla*_KPC_ and in transposable genes upstream and downstream of it (Supplemental Fig. 2). In contrast, the genetic context of *bla*_KPC_ was identical in group YZY. Furthermore, all groups exhibited at least a two-fold decrease in MIC_MEM_ under the influence of carbonyl cyanide m-chlorophenyl hydrazine (CCCP) (Supplemental Table 2).

### The mechanism of carbapenem resistance transformation in missing group

**Fig. 3:**
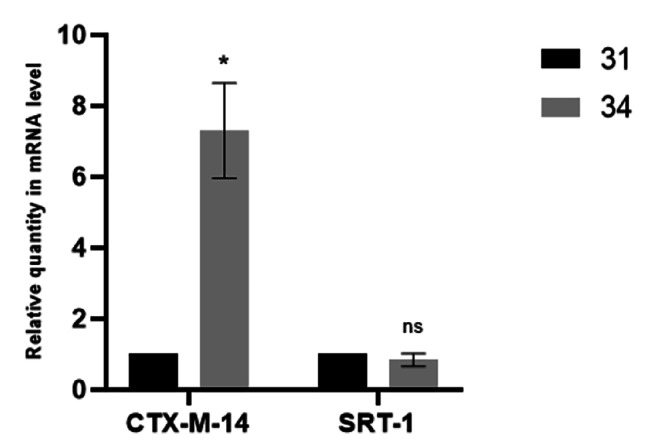
Comparison of *AmpC* gene expression levels in missing group. CTX-M-14: *bla*_CTX−M−14_; SRT-1: *bla*_SRT−1_. *:P < 0.01, ns: not significant

Strains in group LCD (missing group) were separated from different source but shared the same clonal type with the obtaining groups. Despite the absence of carbapenemase-encoding genes, these strains carried two β-lactamase genes, including *bla*_CTX−M−14_, *bla*_SRT−1_ and *bla*_SRT−2_, similar to other strains. However, with a result of BLAST, strain 34 lost a porin gene (Gene ID: 64,177,395) and was more than six times that of strain 31 in *bla*_CTX−M−14_ expression level (Fig. 3).

## Discussion

In this study, we observed the diverse carbapenem resistant transformation mechanism of *S. marcescens* strains. Three patients in seven were stayed in neurosurgery department and two were in intensive care unit during the hospitalization. Besides, only three patients in persisting groups were clinical improved. It was suggested that the selection pressure led to the usage of at least one of the broad-spectrum antibiotics (meropenem, cefoperazone sulbactam and piperacillin tazobactam) in each patient. The transformation mechanism showed a potential relationship with clonal types of each group which indicated the different transformation mechanisms of strains were influenced by the specific genetic background.

In the analysis of horizontal gene transfer process of *bla*_KPC_, various species of carbapenem-resistant strains were observed in the same patient. As demonstrated in Supplemental Fig. 1, we did not find any evidence of gene transferring since none of the strains harboring *bla*_KPC_ gene. Actually, they possessed β-lactam genes including *bla*_OXA_ and *bla*_NDM_. The *bla*_KPC_-harboring plasmids in obtaining groups were IncF types and shared the similar gene context of *bla*_KPC_ with persisting groups. Our work previously confirmed that the pathogenic microorganisms could either being cross-regional transmitted between the environment and humans [21], or being limited to a specific area [22]. Above all, we supposed that the *bla*_KPC_ gene was more likely being transferred from other strains rather than a new clone replacement.

In persisting groups, it was indicated that the mechanism of changed carbapenem resistance was the upregulation of the *bla*_KPC_ expression. With the comparison of the *bla*_KPC_ gene context, the *bla*_KPC_ expression of strain 26 probably upregulated by several gaps. However, mutations of the mobile elements around it which was not determined (Supplemental Fig. 2). No change of the sequence was observed between strains 82 and 152. Because of the significant inhibition effect of CCCP (Supplemental Table 2), the increased MIC_MEM_ of strain 152 might partly attributed to the efflux pump function rather than the upregulated expression of *bla*_KPC_.

In fact, the efflux pumps played a common role in all strains was observed in the present study. As Supplemental Table 2 showed, the MIC_MEM_ of all strains were significantly decreased to a susceptible level. It was proposed that CCCP not only regulated the efflux pumps of carbapenem, but also the expression of *bla*_KPC_, which was a major contributor to carbapenem resistance. As previously reported, the MacB ABC transporter forms a tripartite efflux pump with the MacA adaptor protein and TolC outer membrane exit duct to expel antibiotics from Gram-negative bacteria [23]. Since various mechanisms have been reported, a series of efflux pumps also playing an important role in tigecycline resistance [24–28], but none is confirmed in carbapenem resistance. Till now, only a few efflux systems that belong to different families have been reported for *S. marcescens*. A comprehensive review of efflux systems was conducted, which included a bioinformatical analysis of the genes encoding the RND type systems in *S. marcescens*, based on the homology with the relevant *E. coli* genes [29]. It will promote our understanding of the physiology of the bacteria and detect new molecular mechanisms of resistance.

This was the first exploration of the evolution of drug resistance in CRSM in vivo, according to current studies. This research was restricted in some aspects of the process which necessitate further investigation. One is the regulation of the *bla*_KPC_ that was rarely reported in related literature. To confirm the exact efflux pump target on the specific drug is also a difficult task and required more efforts. Besides, there still has some mechanisms which could be varied by clonal types and have not been detected and mentioned in this study.

The clone replacement does not seem to be the usual means of carbapenem resistance alteration in *S. marcescens*, as it necessitates the bypassing of hand hygiene and maintaining a distance. In clinical practice, antibiotics are the last sword to fight against the infection, but often with a not sharp edge. Thus, the infection control strategy should take the primary and dominant role in preventing the transmission of drug resistance gene. Besides, a better understanding and prediction of resistance patterns of a pathogen will lead to a better selection of active antibiotics for the treatment of multidrug-resistant infections [30]. Therefore, more attention should be paid to the efflux effect of drug research and development in future because of the extensive role in drug resistance.

## Conclusion

*S. marcescens* strains acquired carbapenem-resistance in vivo through the different drug resistance mechanism instead of clone replacing. It also suggested that the specific mechanism of carbapenem resistance was probably related to specific clonal types. These insights into the complex mechanisms underlying carbapenem resistance acquisition have important implications for the development of effective strategies to combat antibiotic resistance in clinical settings.

### Electronic supplementary material

Below is the link to the electronic supplementary material.

Supplementary Material 1

## Data Availability

The sequencing data for the 14 isolates have been deposited at NCBI (BioProject: PRJNA991124) under the accession numbers SAMN36289077-SAMN36289090 (https://www.ncbi.nlm.nih.gov/sra/PRJNA991124).
