# Supplementary material for: A preliminary exploration on the mechanism of the carbapenem-resistance transformation of Serratia marcescens in vivo
Source: BMC Genomics. 2024 Jan 2;25:2. doi: 10.1186/s12864-023-09904-2 (PMC10759614; doi:10.1186/s12864-023-09904-2)
Supplement: Supplementary file 1 — Supplementary Material 1 [file 12864_2023_9904_MOESM1_ESM.docx]

**Supplemental materials**

**
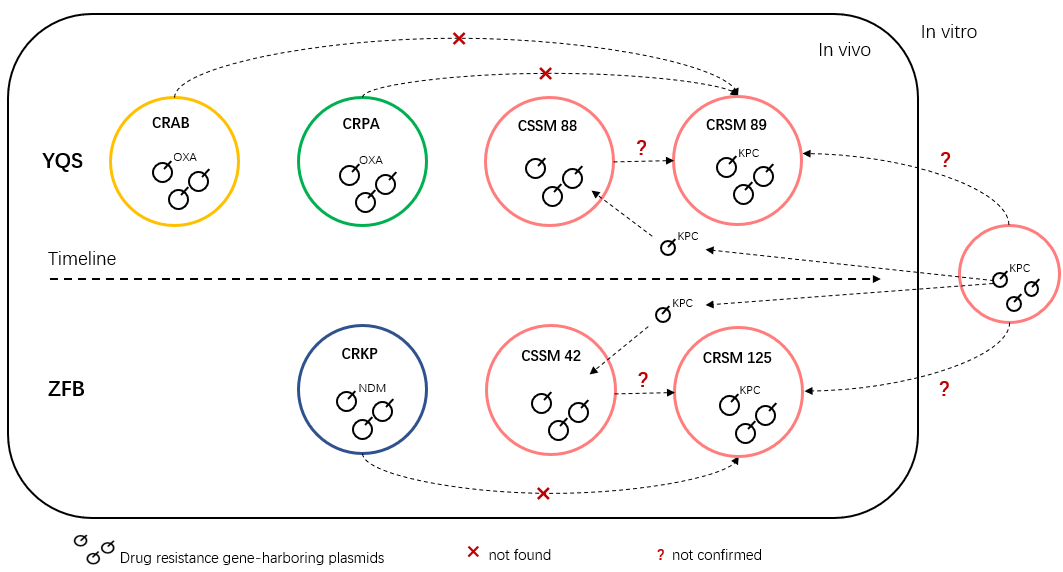
**

**Supplemental Figure 1.** The hypothesis of the *bla*_KPC_ transmission route.

CRSM: carbapenem resistant *Serratia Marcescens*, CSSM: carbapenem susceptible *Serratia Marcescens*, CRAB: carbapenem resistant *Acinetobacter baumannii*, CRPA: carbapenem resistant *Pseudomonas aeruginosa*, CRKP: carbapenem resistant *Klebsiella pneumoniae*.


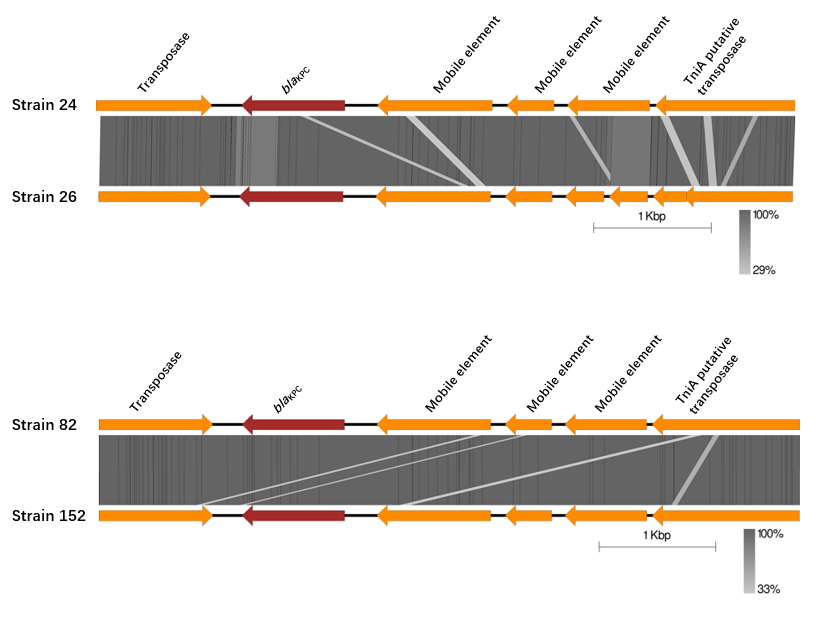


**Supplemental Figure 2.** The genetic context of *bla*_KPC_ in persisting groups FZX and YZY

Figure was created by Easyfig (v2.2.4) software. Arrows present the coding district of genes.

**Supplemental Table 1. The primers used in qPCR .**

| **Genes** | **Primers** | **sequence（5’→3’）** | **Product size（bp）** |
| --- | --- | --- | --- |
| rpoB | rpoB F | TCGAAACGCCTGAAGGTC | 184 |
|  | rpoB R | TTGGAGTTCGCCTGAGC |  |
| *bla*_SRT-1_ | SRT-1 F | GATGATCATGAACGGCACGC | 159 |
|  | SRT-1 R | CACTTGTTGGCCAGCATCAC |  |
| *bla*_CTX-M-14_ | CTX-M-14 F | AACACGTCAACGGCACAATG | 187 |
|  | CTX-M-14 R | ATGGCGGTATTCAGCGTAGG |  |
| *bla*_KPC-2_ | KPC F | TGTCTTGTCTCTCATGGCCG | 123 |
|  | KPC R | TATCCATCGCGTACACACCG |  |

**Supplemental Table 2.** The efflux pumps inhibitor test in seven groups

| **Group** | **Strains NO.** | **MIC_MEM_ (μg/ml)** | **NMP** | **pAβN** | **CCCP (μg/ml)** |
| --- | --- | --- | --- | --- | --- |
| **CMY** | **62** | 0.0625 | 0.0625 | 0.0625 | 0.03125 |
|  | **63** | 4 | 2 | 2 | 0.03125 |
| **FZX** | **24** | 2 | 2 | 2 | 0.03125 |
|  | **26** | 32 | 32 | 32 | 0.03125 |
| **LCD** | **31** | 0.125 | 0.125 | 0.125 | 0.03125 |
|  | **34** | 8 | 8 | 8 | 0.03125 |
| **LAC** | **83** | 0.0625 | 0.0625 | 0.0625 | 0.03125 |
|  | **86** | 8 | 8 | 8 | 0.03125 |
| **YZY** | **82** | 16 | 16 | 16 | 0.125 |
|  | **152** | 32 | 32 | 32 | 2 |
| **YQS** | **88** | 0.0625 | 0.0625 | 0.0625 | 0.03125 |
|  | **89** | 32 | 16 | 32 | 0.0625 |
| **ZFB** | **42** | 1 | 0.5 | 0.5 | 0.03125 |
|  | **125** | 16 | 8 | 8 | 0.03125 |
|  | **ATCC 25922** | 0.03125 | 0.03125 | 0.03125 | 0.0019 |

pAβN: Phe-Arg-β-naphthylamide;NMP: N-methyl-2-pyrrolidone; CCCP: Carbonyl cyanide m-chlorophenyl hydrazone.
